# Detection of Novel Polyomaviruses, TSPyV, HPyV6, HPyV7, HPyV9 and MWPyV in Feces, Urine, Blood, Respiratory Swabs and Cerebrospinal Fluid

**DOI:** 10.1371/journal.pone.0062764

**Published:** 2013-05-08

**Authors:** Rebecca J. Rockett, Theo P. Sloots, Sharleen Bowes, Nicholas O’Neill, Suifang Ye, Jenny Robson, David M. Whiley, Stephen B. Lambert, David Wang, Michael D. Nissen, Seweryn Bialasiewicz

**Affiliations:** 1 Queensland Paediatric Infectious Diseases Laboratory, Queensland Children’s Medical Research Institute, The University of Queensland, Brisbane, Queensland, Australia; 2 Department of Microbiology, Sullivan Nicolaides Pathology, Brisbane, Australia; 3 Immunisation Program, Communicable Diseases Branch, Queensland Health, Herston, Australia; 4 Departments of Molecular Microbiology and Pathology & Immunology, Washington University School of Medicine, St. Louis, Missouri, United States of America; 5 Microbiology Division, Pathology Queensland Central Laboratory, Herston, Queensland, Australia; University of Kansas Medical Center, United States of America

## Abstract

Eight novel human polyomaviruses have been discovered since 2007. Prevalence rates and tissue tropism for the most recent members HPyV 6, 7, 9, TSPyV and MWPyV are largely unknown. We used real-time PCR to determine the presence of HPyV 6, 7, 9, TSPyV and MWPyV in feces (n = 263), urine (n = 189), blood (n = 161), respiratory swabs (n = 1385) and cerebrospinal fluid (n = 171) from both healthy control children and children and adults undergoing diagnostic testing. Whole genome sequencing was able to be performed on 9 MWPyV positive specimens. Novel polyomaviruses were only detected in respiratory swabs and feces, with no detections of HPyV 9 in any sample type. MWPyV was found to be the most prevalent novel polyomavirus, being detected in 18 (1.5%) respiratory specimens from symptomatic patients, 16 (9.8%) respiratory sample from healthy control children, 11 (5.9%) fecal specimens from patient suffering gastrointestinal illness, and in 13 (15.3%) of feces from healthy control children. MWPyV was found only in respiratory and fecal specimens from children, the oldest being 9 years old. HPyV 6, 7, 9 and TSPyV were also detected in respiratory specimens and fecal specimens at low prevalence (<1.3%). The majority of these detections were found in immunocompromised patients. Our findings suggest that MWPyV can result in a subclinical infection, persistent or intermittent shedding, particularly in young children. The other novel polyomaviruses were also found in respiratory and fecal specimens, but at lower prevalence and most commonly in immunocompromised individuals.

## Introduction

In the last five years, eight new human polyomaviruses have been discovered, predominantly through the use of next generation sequencing techniques. In 2007, the first two novel polyomaviruses, WU and KI were identified in children suffering from acute respiratory tract infections. [1], [2] A year later, Merkel cell polyomavirus (MCPyV) was identified in Merkel cell carcinoma (MCC) tissue, with strong evidence suggesting MCPyV genome integration triggers cell transformation in MCC. [3] In 2010, human polyomaviruses 6 (HPyV 6) and 7 (HPyV 7) were discovered on the foreheads of healthy volunteers. [4] In the same year another skin-associated polyomavirus, Trichodysplasia spinulosa-associated polyomavirus (TSPyV) was isolated from the face of an immunocompromised patient suffering from Tricodysplasia spinulosa. This condition is found exclusively in immunocompromised patients and leads to numerous follicular extensions around the face and nose. [5] In 2011, human polyomavirus 9 (HPyV 9) was amplified from the blood sample of a renal transplant patient using degenerative primers, [6] and in 2012 Malawi polyomavirus (MWPyV) was identified in a stool sample of a healthy child. [7].

Recently, two other polyomaviruses were reported, provisionally named HPyV10 and MXPyV. HPyV10 was isolated from condylomas on the buttocks of patients suffering a rare genetic disorder known as warts, hypogammaglobulinemia, infections, and myelokathexis (WHIM) syndrome. [8] MXPyV, like MWPyV, was identified in stool samples from children suffering diarrhea in Mexico. [9] The whole genomes reported for these viruses share at least 94.6% sequence identity with the index MWPyV sequence (MA095).

The first human polyomaviruses JC (JCPyV) and BK (BKPyV) were discovered in 1971 and are well characterized, with JCPyV and BKPyV infection generally occurring in early childhood resulting in sub-clinical or a mild respiratory infection.[10]–[12] After primary infection a low persistent infection is thought to continue, possibly in the lymphoid, neuronal, kidney or hematopoietic tissues.[13]–[15] This persistent infection can reactivate in immunocompromised patients causing, in some cases, severe clinical symptoms. Progressive Multifocal Leukoencephalopathy (PML) occurs when JCPyV reactivates in immunocompromised patients, causing a lytic infection of the oligodendricytes leading to demyelisation within the brain that rapidly progresses to a fatal outcome. [10], [16] In bone marrow (BMT) and renal transplant patients, reactivation of BKPyV in urogenital tissues may cause hemorrhagic cystitis (HC) and nephropathy, which can lead to graft rejection in up to 10% of kidney transplant recipients. [17], [18].

Numerous studies have investigated prevalence rates, basic epidemiology, genome variability and the possible pathogenicity of WUPyV, KIPyV and MCPyV,[19]–[27] however as yet little similar data are available for HPyV 6,7,9, TSPyV and MWPyV infections.[7]–[9], [28] Given the potentially serious implications of polyomavirus infections in immunocompromised populations, and the possible role of these viruses in cell transformation, it is important to further understand the biology and prevalence of HPyV 6,7,9, TSPyV and MWPyV. In this study, we used real-time PCR and DNA sequencing to investigate the presence of these novel viruses in a variety of specimen types collected from different patient populations.

## Materials and Methods

### Samples

An overview of specimens tested and associated basic demographic information is outlined in Table 1. Briefly, Blood (n = 161), urine (n = 189), CSF (n = 171), feces (n = 263) and respiratory samples (n = 1385) from a number of existing specimen banks were tested retrospectively. These included specimens obtained from healthy volunteers and symptomatic patients undergoing routine diagnostic testing at Pathology Queensland, Australia.

**Table 1:** Study sample population details and their associated MWPyV, HPyV 6, 7 &9 and TSPyV detections.

Sample type	Disease state	Age range (mean; median; age unknown)	MWPyV n (%)	HPyV 6 n (%)	HPyV 7 n (%)	TSPyV n (%)	HPyV 9 n (%)
Respiratory n = 1232	Symptomatic	0.02–92.4 years(16.6; 1.7; 36)	18 (1.5)	2 (0.16)	3 (0.24)	1 (0.08)	0
Respiratory n = 153	Healthy	0–17.2 years(4.8; 2.1; 3)	14 (9.2)	0	0	0	0
Blood n = 161	Symptomatic	1.2–77.3 years(45.4; 49.97; 10)	0	0	0	0	0
CSF n = 171	Symptomatic	0–92.6 years(28.0; 23.17; 1)	0	0	0	0	0
Urine n = 189	Symptomatic	0.03–94.8 years(19.1; 16.3; 6)	0	0	0	0	0
Feces n = 185	Symptomatic	0.07–94.8 years(22.4; 4; 66)	9 (4.9)*	1 (0.5)	1 (0.5)	1 (0.5)	0
Feces n = 78	Healthy	0–7.46 years(1.4; 1.2; 3)	10 (12.8)	0	0	1 (1.2)	0

* 11 positive specimens collected, three from one patient tested three times over 5 days therefore only one positive specimen was used to calculate prevalence.

#### Respiratory samples

The respiratory specimens (n = 1232) examined in this study were obtained from 1143 patients with respiratory symptoms which had diagnostic specimens submitted for testing to Pathology Queensland, Australia, between October 2006 and December 2008. The majority of these samples were collected in 2008 with approximately equal numbers of specimens from each month (80 samples per month). The respiratory specimens consisted of 993 nasopharyngeal aspirates (NPA), 145 Bronchoalveolar lavage (BAL), 31 nose and throat swabs, 33 bronchial washing, 6 sputum, 2 lung tissues and 22 respiratory specimens of unknown type. Specimens were from both immunocompetent and immunocompromised adult and pediatric patients (age range 0.02–92.4 years, mean 16.6 years, median 1.7 years and 36 with age not recorded). These patients presented with symptoms consistent with both upper and lower respiratory tract infection. These samples had previously been tested for common respiratory viruses by routine diagnostic PCR – influenza A and B (INF A & B), respiratory syncytial virus (RSV), parainfluenza viruses 1, 2, 3, & 4 (PIV 1, 2, 3 & 4), adenoviruses, human metapneumovirus (HMPV), human rhinoviruses (HRV) and human bocavirus (HBoV) as previously described.[29]–[33].

#### Blood samples

Blood samples were obtained from 161 patients undergoing routine BKPyV, cytomegalovirus, or adenovirus testing, during October 2006 to December 2009. These patients were aged 1.2–77.3 years (mean 45.42 years, median 49.97 and 10 of unknown age), comprising 125 immunocompromised patients and 36 immunocompetent patients.

#### CSF samples

CSF samples were from 171 patients undergoing microbiological testing from March 2010 until December 2010 for suspected neurological disease. These patients were aged 0–92.6 years (mean 27.97, median 23.17, and 1 of unknown age).

#### Urine samples

Urine samples were from 189 patients undergoing testing from January 2009 until September 2012. These patients were aged between 0.03–62.5 years (mean 19.08, median 16.33 and 6 of unknown age).

#### Fecal samples

Fecal specimens were collected from 185 patients with gastrointestinal illness and tested by antigen-based assays for both rotavirus and adenovirus by Pathology Queensland or Sullivan and Nicolaides Pathology, Brisbane, Australia, during February 2011 until March 2012. Patients were aged between 0.07–94.8 years (mean 22.4 years, median 4.4 years and 66 specimens were from patients of unknown age).

#### Healthy control sample population

Two separate healthy control sample populations were used in this study. The first, healthy control sample population A comprised nasopharyngeal aspirates from 75 healthy children without recent respiratory symptoms, undergoing endoscopy or colonoscopy. Specimens were collected between June 2007 and February 2011. The children ranged in age from 0.2–17.2 years (mean 8.3 years, median 8.4 years and 0 of unknown age).

Healthy control sample population B consisted of paired throat swabs and fecal specimens from 78 otherwise healthy children recruited from the Emergency Department (with a non-infectious presentation) or child care centers near the Royal Children’s Hospital, Brisbane. Specimens were collected from September 2007 until June 2010, with children’s ages ranging from 0–7.46 years (mean 1.4 years, median 1.2 years and 3 of unknown age).

Both control populations excluded children with symptoms of respiratory or gastrointestinal infections in the seven days prior to collection. A follow-up phone call was made approximately 4 days after specimen collection to record any respiratory or gastroenteritis symptoms post-specimen collection (follow-up clinical information is not available for 17 participants in healthy control population A and four participants in healthy control population B). If respiratory or gastrointestinal symptoms were recorded in the follow-up phone call, specimens where excluded from the healthy control populations (six participants excluded from healthy control population A and seven participants excluded from healthy control population B).

### Sample Processing

Total nucleic acids were extracted from 0.2 mL of blood, urine and CSF using the QIAxtractor instrument and DX viral nucleic acid reagents (Qiagen, Australia) according to manufacturer’s instructions. To monitor the efficiency and reproducibility of DNA extraction, samples were spiked with 10^4^ copies of equine herpes virus (EHV) DNA (PCR crossing point of 30 cycles) before DNA extraction. [34] Samples providing a crossing point within 3 cycles of 30 cycles were considered efficiently extracted and free of PCR inhibitors. Samples failing this procedure were re-extracted.

Additional processing was required for respiratory swabs and fecal specimens before extraction. Briefly, 2.0 mL of phosphate buffered saline was added to each collection tube containing the respiratory swab and vortexed vigorously before 0.2 mL was removed for extraction. A 10 µl loop was used to create a fecal suspension 1 mL of PBS, which was then vortexed and clarified by centrifugation at 6,000 g before 0.2 mL was removed for extraction.

To reduce testing costs, nucleic acids from each specimen type were combined in 10×10 pools for initial screening as previously described. [35] Individual specimens in polyomavirus positive pools were retested with a second assay specific for each polyomavirus. This secondary assay was designed in a different region of the polyomavirus genome. For specimens to be considered polyomavirus positive, both assays had to produce a positive amplification curve below 41 cycles. Limited sequence data is currently available for the novel polyomaviruses tested for in this study therefore some virus positive samples may potentially have been missed due to sequence variation within primer and probe targets.

### Virus Detection

Pooled sample extracts were screened by real-time polymerase chain reaction (rtPCR) assays for HPyV6, [36] HPyV7, [6], [36] TSPyV [5] and HPyV9 in a duplex format (Duplex format; HPyV6A+HPyV9B and HPyV7E+TSPyVA) using previously published assays, with primer and probes for each assay shown in Table 2.

**Table 2:** Primer and probes used in screening and confirmatory rtPCR assays.

Assay	Target	Primer (5′ –3′)
MW-E forward	VP1	CATTGATGGACAGCCAATGG
MW-E reverse	VP1	TCCTGGAAGAGGTTCTGTTCCTT
MW-E probe	VP1	TGGGACTGATAATCAAGTACAGGATGTAACTGTGT
ES105 forward [7]	LTag	TGAGAAGGCCCCGGTTCT
ES106 reverse	LTag	GAGGATGGGATGAAGATTTAAGTTG
ES107 probe	LTag	CCTCATCACTGGGAGC
HPyV 6A forward [36]	LTag	ACCAGGTGGGTGATGAAGACA
HPyV 6A reverse	LTag	CGCCTGAATGTTTTAAAGGAGAA
HPyV 6A probe	LTAg	AGGAAGATGCCTTGTCACAGAAAAGGAAATG
HPyV 6D forward [36]	LTAg	TTGAGGAGCTGGACAAAGAGATT
HPyV 6D reverse	LTAg	TCTGGGAAGCTTTTGAATTGGT
HPyV 6D probe	LTAg	AGGAAGATGCCTTGTCACAGAAAAGGAAATG
HPyV 7E forward [36]	LTAg	AAGACATTCAGTCTTTGCATTTTCTG
HPyV 7E reverse	LTAg	CCCCTCATACAGCATAAGGTTAGATT
HPyV 7E probe	LTAg	CCACCTTTATCTGGATGATACTTTTTGCTGGC
HPyV 7D forward [36]	VP2/3	GAGGAAGGAAACACTCCCCAGTA
HPyV 7D reverse	VP2/3	TTCACTTCTTTTTGTAGCTCCTCAAG
HPyV 7D probe	VP2/3	ACTATACCTCAATGGATGCTTTTTGT
TSPyV A forward [5]	LTAg	TGTGTTTGGAAACCAGAATCATTTG
TSPyV A reverse	LTAg	TGCTACCTTGCTATTAAATGTGGAG
TSPyV A probe	LTAg	TTCTTCTTCCTCCTCATCCTCCACCTCAAT
TSPyV B forward [5]	VP1	AGTCTAAGGACAACTATGGTTACAG
TSPyV B reverse	VP1	ATTACAGGTTAGGTCCTCATTCAAC
TSPyV B probe	VP1	ACAGCAGTGACCAGGACAAGCCTACTTCTG
HPyV 9 A forward	VP1	CTAGGGAACAATTTGAATATCAGGAA
HPyV 9 A reverse	VP1	ATAGTGTCCAGATCTAGGCTCTGAAC
HPyV 9 A probe	VP1	AAGTTAGGCTGAGGCGGGAGATAGGG
HPyV 9 B forward	LTAg	CTAGGGAACAATTTGAATATCAGGAA
HPyV 9 B reverse	LTAg	ATAGTGTCCAGATCTAGGCTCTGAAC
HPyV 9 B probe	LTAg	AAGTTAGGCTGAGGCGGGAGATAGGG

The HPyV9 testing was performed using two novel rtPCR assays designed for this study. The HPyV9B assay the large T-antigen, and was duplexed with the HPyV6A assay for sample pool testing; HPyV9B assay targeted targeted VP1and was used to test HPyV9A positive pools. These assays were designed using Primer Express 2.0 software and HPyV9 sequences publicly available on Genebank (accession HQ696595 accessed on 06 November 2012).

MWPyV testing was performed using two rtPCR assays. These comprised a previously described method by Siebrase *et al* 2012 (MW ES107) [7] and a newly designed assay (MW-E) developed as part of this study, targeting a conserved region of the MWPyV VP1 gene (Table 2). Primers and probes for MW ES107 and MW-E also have 100% homology to the VP1 sequences reported for HPyV10 and MXPyV.

Briefly, the MW-E assay was designed using Primer Express 2.0 software and the two MWPyV whole genome sequences publicly available on Genebank (accessions; MA095, NC_018102, WD976, JQ898292.1 accessed on 08 August 2012). Analytical sensitivities for the three assays developed as part of this study (MW-E, HPyV9A and HPyV9B) were <10 copies/ul of extract. A common reaction mix was used for all polyomavirus rtPCR assays. This consisted of 12.5 µl of Quantitect Probe PCR Mix (Qiagen, Australia), 10 pmol of each primer, 4 pmol of each probe and 5.0 µl of template to make a 25.0 µl final reaction volume. The rtPCR was performed on the Rotorgene 6000 or Rotorgene Q (Qiagen, Australia) under the following conditions: 15 min incubation at 95°C, followed by 45 cycles of 95°C for 15sec and 60°C for 1 min.

### DNA Sequencing

Whole genome sequencing was performed using a series of walking primers. Sequence data were aligned and manipulated with Bioedit Sequence Alignment Editor version 7.0.9.0. [37] Phylogenetic analysis was performed using a neighbour-joining (NJ) analysis with 1000 bootstrap and the Tamura-Nei substitution model in the (Mega 4.1) software package. [38] Whole genome sequences produced from this study were compared to the four published MWPyV whole genomes (accessions; MA095 NC_018102, WD976 JQ898292.1, HPyV10 JX262162.1, UC-MXPyV JX259273.1 accessed on 20 November 2012).

### Data Analyses and Approval

Statistical comparisons were performed using the two-tailed fisher’s exact test.

This study, including the use of specimens from a number of sources, was approved by the Queensland Children’s Health Services Human Research Ethics Committee. Written informed consent was obtained from the guardians of the healthy control populations for extended virus testing. We received a waiver from the need to obtain individual consent from the Ethics Committee, in accordance with National Guidelines [39], for patients whose stored specimens, originally submitted for routine diagnostic testing, were used in this study.

## Results

The results for HPyV 6, 7, 9, TSPyV and MWPyV screening in the various sample types are presented in Table 1 and Table 3. Polyomavirus detections were only found in respiratory and fecal specimens, with the most frequently detected polyomavirus being MWPyV. HPyV 9 was not detected in any specimen.

**Table 3:** Individual polyomavirus detections for symptomatic patients, including available demographic information, PCR Ct values, viral co-detections and clinical information.

Symptomatic respiratory specimens							
Polyomavirus	Sample	Age (years)	Sex		Ct value	Co-detections	Clinical information
MWPyV	NPA	4.21	M		28.87	HRV, HBoV	ALL
MWPyV	NPA	1.22	F		29.1		
MWPyV	NPA	3.80	M		29.27	PIV 3,HRV	ALL
MWPyV	NPA	2.01	M		29.17	Adenovirus, HRV	Conjunctivitis
MWPyV	NPA	0.54	F		30.81	HRV	Gastroschisis, TPN dependent
MWPyV	NPA	1.35	F		30.94	RSV, HRV	Cough, diarrhea
MWPyV	NPA	6.64	F		30.59	hMPV	ALL
MWPyV	NPA	0.60	F		31.5	hMPV, HRV	Yellow rhinorhea
MWPyV	NPA	1.71	M		33.13	HRV	
MWPyV	NPA	4.38	F		32.75	HRV	ALL
MWPyV	NPA	1.36	M		34.65	Adenovirus	Febrile, cough, increased respiratory effort
MWPyV	Aspirate	1.81	M		34.8	PIV 1, HRV, HBoV	2 days post unexplained seizures
MWPyV	NPA	2.85	F		35.89	hMPV	Febrile for 2+ days
MWPyV	NPA	1.09	M		38.09	HRV	Febrile convulsions
MWPyV	NPA	0.14	M		35.19	PIV 3	Bronchiolitis
MWPyV	NPA	1.77	M		37.11	PIV 3 & 4, Adenovirus	PUO Febrile
MWPyV	NPA	1.39	M		38.8	HRV	CP, recurrent RTIs, respiratory distress, wheeze, rhinorrea
MWPyV	NPA	5.03	M		35.57		Sotos syndrome, cough
TSPyV	NPA	0.57	M		37.86	hMPV	
HPyV6	Lavage Bronchus/RLL	48.2	M		39.17		Pre-BMT, Neutropenic
HPyV6	NPA	61.4	F		28.92		HIV
HPyV7	NPA	1.04	M		36.84	Adenovirus	Neutropenic, fevers
HPyV7	NPA	67.2	M		35.9		AML
**Symptomatic fecal specimens**							
**Polyomavirus**	**Sample**	**Age (years)**	**Sex**		**Ct value**	**Co-detections**	**Clinical information**
HPyV7	BAL	13.6	F		32.83		BMT
MWPyV	Fecal	1.01	M	29.52		Rotavirus	Diarrhea, cough
MWPyV	Fecal	1.46	F	25.65		Rotavirus	Chronic diarrhea
MWPyV	Fecal	n/a	n/a	33.67		Rotavirus	n/a
MWPyV	Fecal	n/a	n/a	33.76		Rotavirus	n/a
MWPyV	Fecal	n/a	n/a	30.25		Adenovirus	n/a
MWPyV	Fecal	n/a	n/a	32.13		Adenovirus	n/a
MWPyV	Fecal	n/a	n/a	35.76		Adenovirus	n/a
MWPyV	Fecal	n/a	n/a	33.44		Rotavirus	n/a
*MWPyV	Fecal	1.21	M	29.58			ALL
*MWPyV	Fecal	1.21	M	30.71			ALL
*MWPyV	Fecal	1.20	M	28.99			ALL
TSPyV	Fecal	14.4	F	31.77			BMT/ALL
HPyV6	Fecal	n/a		36.68		Rotavirus	n/a
HPyV7	Fecal	63.8	F	36			Renal transplant

* Same patient collected over 5 days.

ALL – acute lymphoblastic leukaemia, AML – acute myeloid leukaemia, BMT – bone marrow transplant, TPN – total parenteral nutrition, CP – cerebral palsy, PUO – pyrexia of unknown origin, URTI – upper respiratory tract infection, HIV – human immunodeficiency virus.

### Polyomavirus Detections - Respiratory Specimens

In respiratory specimens, 18 (1.5%) of samples from the symptomatic patients and 14 (9.2%) from the healthy control children were positive for MWPyV (Table 1). All MWPyV positive subjects were aged less than 10 years. Nearly all of the MWPyV positive diagnostic specimens (16/18) had at least one respiratory pathogen co-detected with MWPyV (Table 3); 9/16 had one co-detection, 5/16 had two other viruses co-detected and 2/16 had three other pathogens co-detected with MWPyV. Clinical symptoms were available for 11 of the 18 MWPyV positive patients. These included fever, febrile convulsions, cough, wheeze, rhinorrhea, diarrhea, conjunctivitis and increased respiratory effort. Four MWPyV positive respiratory specimens where from immunocompromised children, all diagnosed with acute lymphoblastic leukaemia (ALL) (Table 3).

For the remaining polyomaviruses, HPyV6, 7 and TSPyV were detected in 2 (0.16%), 3 (0.24%), and 1 (0.08%) of diagnostic respiratory samples, respectively. HPyV 6, 7 and TSPyV were not detected in any respiratory specimens from the healthy children sample population. The two respiratory specimens that were positive for HPyV6 were both from adults and comprised a BAL specimen from a male aged 48 years and a NPA from a female aged 61 years. Both HPyV6-positive patients were immunocompromised; the female patient was neutropaenic and pre-BMT and the male patient was HIV positive. In both of these patients HPyV6 was the only virus detected in each specimen.

The three respiratory specimens that were positive for HPyV7 were from two symptomatic children and one immunocompromised adult male. The symptomatic HPyV 7 positive children were a BAL specimen from a 1 year old male with neutropaenic fevers, and a BAL from a 13 year old female BMT recipient. The positive sample from the adult was an NPA from an immunocompromised male 67 years of age, suffering from acute myleiod leukaemia (AML). These samples were positive for HPyV7 only, except for the sample from the 1 year old male, in which adenovirus was also detected.

### Polyomavirus Detections - Fecal Specimens

MWPyV positive fecal specimens comprised 11 (4.9%) symptomatic fecal specimens and 10 (12.8%) specimens from the healthy control group. Three of the symptomatic positive fecal specimens were taken from the same patient over a five day period; this was recorded as a single positive for prevalence calculations. MWPyV-positive patients where from children aged between 1 and 2 years, 2 months of age (6 patients of unknown age). In the symptomatic specimens, 8/11 (72.7%) reported a positive co-detection with either rotavirus or adenovirus (Table 3).

A single positive specimen was detected for each of HPyV6, 7 and TSPyV in the symptomatic sample population. No clinical information was available for the HPyV6 positive patient. The HPyV7 positive patient was female, 63 years of age, and a renal transplant recipient. The TSPyV positive patient was female, aged 14 years, had ALL, and was a BMT recipient. One specimen was positive for TSPyV in the healthy control sample population; a one year old male (Table 4).

**Table 4:** Individual polyomavirus detections for healthy control populations, including match fecal and respiratory specimens.

Healthy control population A					
Polyomavirus	Sample	Age (years)	Sex	Ct value respiratory	Ct value Fecal
MWPyV	NPA	0.9	F	30.61	n/a
MWPyV	NPA	9.7	M	34.03	n/a
MWPyV	NPA	9.5	F	35.28	n/a
MWPyV	NPA	1.1	M	35.98	n/a
MWPyV	NPA	1	M	36.87	n/a
**Healthy control population B**					
**Polyomavirus**	**Sample**	**Age (years)**	**Sex**	**Ct value respiratory**	**Ct value Fecal**
MWPyV	Nose and throat swab/fecal	2.09	M	30.54	34.77
MWPyV	Nose and throat swab/fecal	1.76	M	29.99	30.95
MWPyV	Nose and throat swab/fecal	1.2	M	31.96	33.33
MWPyV	Nose and throat swab/fecal	1.76	M	34.6	ND
MWPyV	Nose and throat swab/fecal	1.03	F	33.88	ND
MWPyV	Nose and throat swab/fecal	1.48	M	35.64	33.72
MWPyV	Nose and throat swab/fecal	0.84	M	35.83	ND
MWPyV	Nose and throat swab/fecal	1.45	M	34.31	ND
MWPyV	Nose and throat swab/fecal	1.31	M	ND	32.58
MWPyV	Nose and throat swab/fecal	0.64	M	ND	32.68
MWPyV	Nose and throat swab/fecal	1.17	F	ND	33.62
MWPyV	Nose and throat swab/fecal	1.62	F	ND	31.01
MWPyV	Nose and throat swab/fecal	1.71	M	ND	33.34
MWPyV	Nose and throat swab/fecal	1.21	F	ND	32.62
TSPyV	Nose and throat swab/fecal	1.32	M	ND	36.35

ND – Not detected.

MWPyV detection was significantly (p = 0.0001) more common in children younger than five years (34/1017) than in children aged five years and older (4/425).

A total of 26 MWPyV positive specimens were detected in healthy control populations (n = 217) which was significantly higher than the 29 detections in symptomatic specimens (n = 1417) using a two-tailed Fishers exact test (p = 0.0001).

### Healthy Control Population B - Paired Respiratory and Fecal Specimens

Control population B contained paired fecal and respiratory specimens from 78 healthy children. MWPyV was detected in both the fecal and respiratory specimens of 4 participants. In addition, MWPyV was detected in 4 participants’ respiratory swabs, but not in the paired fecal specimens, and in the fecal specimen but not the respiratory specimen of 7 participants (Table 4).

### MWPyV Whole Genome Sequencing

**Figure 1:**
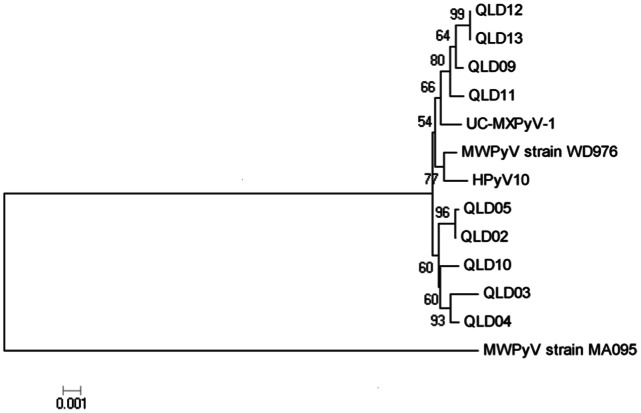
Neighbour-Joining phylogeneic analysis of 13 MWPyV whole genome sequences (GenBank accession numbers: MA095 NC_018102, WD976 JQ898292.1, HPyV10 JX262162.1, UC-MXPyV JX259273.1 QLD02 KC549586, QLD03 KC549587, QLD04 KC549588, QLD05 KC549589, QLD09 KC549590, QLD10 KC549591, QLD11 KC549592, QLD12 KC549593, QLD13 KC549594).

Whole MWPyV genome sequence data were generated for only nine of the MWPyV-positive samples (GenBank accession numbers: KC549586, KC549587, KC549588, KC549589, KC549590, KC549591, KC549592, KC549593, KC549594). Although a total of 51 MWPyV positives samples were detected in this study, the cycle threshold values for the majority of these were high (average Ct value of 33, range 24.91–40.37). This is consistent with low viral loads which are difficult to sequence reliably, and we were unable to obtain quality sequence data over the entire viral genome. A phylogenetic tree, including the four previously published MWPyV whole genome sequences, is shown in Figure 1. Overall homology between all 13 MWPyV genome sequences ranged from 95.6 to 100%. Sequence homology of greater than 99.6% was seen between all sequences except the index case (MA095) from Malawi (95.6%). Notably, an 11 base pair insertion (nucleotide position 171–181) within the non-coding control region was noted in all sequences except the index case MA095.

## Discussion

We used rtPCR to measure the prevalence of five novel human polyomaviruses, HPyV 6, 7, 9, TSPyV and MWPyV in blood, urine, CSF, respiratory and fecal specimens from both healthy and symptomatic populations. Unlike JCPyV and BKPyV, no evidence was found of these novel polyomaviruses in blood, urine or CSF. [19] In respiratory samples, MWPyV was found to be the most prevalent novel polyomavirus; detected in 1.5% of respiratory specimens from symptomatic patients and 9.2% of respiratory specimens from our young healthy control children. This differs from a previous study from Mexico showing a detection rate of 0.74% in nasal washes from children suffering respiratory infections. [9].

We detected MWPyV in 4.4% of fecal specimens collected from patients suffering gastroenteritis and 12.8% in healthy control children, which is higher than the previously reported rates of 2.3%, 3.3%, and 0% from children with diarrhea in St Louis, Missouri, [7] California (unspecified number of stool samples from children not suffering diarrhea) and Chile. [9] In contrast to our findings however, Yu *et al* (2012) reported a high detection rate of 12.5% in children suffering diarrhea in Mexico. We also detected HPyV6, 7 and TSPyV in respiratory and fecal specimens, but at low prevalence (<1%), confirming very recent publications that reported HPyV6, 7, 9 and TSPyV in respiratory, urine and fecal specimens from immunocompromised children in St Louis, Missouri. [28] HPyV9 was not detected in any of our samples. HPyV9 has been reported in the blood of renal transplant recipients, and our inability to detect HPyV9 may reflect the relatively small number of blood samples from immunocompromised subjects in our study (n = 125).

Detection of MWPyV exclusively occurred in children younger than 10 years of age, which follows the generally accepted paradigm of polyomavirus infection occurring early in life. It is therefore not surprising that children younger than five years of age had significantly higher MWPyV detection rates than children older than five years and adults.

Interestingly, detection of MWPyV was significantly higher in the fecal and respiratory specimens of healthy children compared to the symptomatic sample populations. This trend was also seen in age matched control populations from children in Chile where 4.2% of control specimens were MWPyV positive, but none in samples from children with diarrhea. It appears that infection with MWPyV in young children can be asymptomatic, and given that the majority (16/18) of MWPyV-positive respiratory specimens from symptomatic patients were co-detected with a least one other respiratory pathogen, many identifications may be coincidental. Likewise in our study, 8/11 MWPyV-positive diagnostic fecal specimens were co-detected with either adenovirus or rotavirus. This is similar to the high co-detection rates reported for WUPyV and KIPyV infections in respiratory samples, and raises questions about the clinical significance of these viruses in childhood acute infectious disease. [40] Nonetheless, the detection of MWPyV in matched fecal and respiratory specimens from healthy children may suggest a fecal-oral mode of transmission for MWPyV, as proposed for other human polyomaviruses. [41].

Currently there is very little known about sequence variation of MWPyV globally. The Australian isolates from this study clustered with the three existing isolates from the United States and Mexico. The isolate from the index case originating in Malawi was divergent from the Australian, US, and Mexican isolates. Notably, sequence variation between the index case and the Australian isolates was 5.3%, whereas only 0.4% variation was observed between the Australian and North American isolates. While these data are limited, they are suggestive of a geographical distribution for MWPyV similar to that of BKPyV. Previous studies have shown that associations between BKPyV genotypes and geographical locations (America, Asia, and Europe) are sufficiently consistent that they can be used to trace human migration. [42] It is likely that the high sequence homology observed for MWPyV strains from Australia and the US may be because the samples were from individuals of European descent having a common ancestry. [42], [43] An 11 bp insertion has been observed in the non-coding control region of the North American MWPyV positive isolates. [7], [9] This insertion was also found in all the Australian isolates. While the significance of this insertion is yet to be determined, it is similar to insertions reported within the KIPyV non-coding control region and it is postulated that may have some effect on transcription factor binding sites. [44].

HPyV6, 7 and TSPyV were detected in both children and adults, albeit at low prevalence. However, all detections in adults were from immunocompromised patients. Interestingly, a recent study of immunocompromised children also found a low prevalence of HPyV6, 7, 9 and TSPyV in respiratory, fecal and urine specimens collected longitudinally. [28] Previous studies have hypothesised an increased prevalence of WUPyV and KIPyV in immunocompromised populations, however most have failed to demonstrate either an increased incidence or more severe clinical symptoms for this patient group.[45]–[48] It should also be noted that HPyV 6, 7 and TSPyV have all been detected on human skin of healthy subjects, and that the possibility of contamination from the skin during collection of the respiratory and fecal samples must be considered.

### Conclusions

In summary, the results show that MWPyV, but not HPyV 6, 7, 9, or TSPyV, can be detected occasionally in respiratory and fecal specimens submitted for viral investigations. However, the high rate of detection of MWPyV in samples collected from asymptomatic age-matched controls confounds the interpretation of the role of this virus in human disease. Like WUPyV and KIPyV, a clear role in disease is not evident but further studies of samples, particularly from immunocompromised patients, are warranted. Our results also demonstrate that MWPyV may be shed simultaneously both within the gastrointestinal and respiratory tracts and may be indicative of a possible fecal-oral route of transmission during early childhood.
